# Single-Coil Eddy Current Sensors and Their Application for Monitoring the Dangerous States of Gas-Turbine Engines

**DOI:** 10.3390/s20072107

**Published:** 2020-04-08

**Authors:** Sergey Borovik, Yuriy Sekisov

**Affiliations:** Institute for the Control of Complex Systems of Russian Academy of Sciences, Samara 443020, Russia; sekisov@iccs.ru

**Keywords:** gas-turbine engine, blade tips, radial clearance, displacement measurement, bearing assembly, oil debris monitoring, single-coil eddy current sensor, measuring system, test bench, experimental studies

## Abstract

The creation and exploitation of gas turbine engines (GTE) often involve two mutually exclusive tasks related to ensuring the highest reliability while achieving a good economic and environmental performance of the power plant. The value of the radial clearance between the blade tips of the compressor or turbine and the stator is a parameter that has a significant impact on the efficiency and safety of the GTE. However, the radial displacements that form tip clearances are only one of the components of the displacements made by GTE elements due to the action of power loads and thermal deformations during engines’ operation. The impact of loads in conjunction with natural aging is also the reason for the wear of the GTE’s structural elements (for example, bearing assemblies) and the loss of their mechanical strength. The article provides an overview of the methods and tools for monitoring the dangerous states of the GTE (blade tips clearances, impellers and shafts displacements, debris detecting in lubrication system) based on the single-coil eddy current sensor, which remains operational at the temperatures above 1200 °C. The examples of practical application of the systems with such sensors in bench tests of the GTE are given.

## 1. Introduction

The high-performance gas turbine engine (GTE) is the basis for creating promising equipment for aviation, land and marine applications. It is known that the main energy, strength, economic and environmental indicators of the GTE are largely determined by the value of the radial clearances (RC) between the blade tips and the stator of the compressor and turbine of the GTE. It is noted by Danilchenko et al. [1] that the dependence of the efficiency value on the RC is nonlinear, and at high-pressure compressor stages, when the RC increases by 1%, the efficiency can decrease by 2%, and the coefficient of aerodynamic stability margin can decrease by 3%. Melcher et al. [2], with reference to [3,4,5], mention the empirical rule according to which a decrease in turbine clearances by 0.25 mm (0.010 in) leads to a decrease in exhaust gas temperature by 10 °C (18 °F) and an increase in turbine efficiency by 1%. As a result, the specific fuel consumption is reduced by 1% and the emission of harmful substances is reduced proportionally. The same source claims that despite the seemingly insignificant effect of reducing the specific fuel consumption, in financial terms, this leads to annual savings of more than $160 million in prices at the time of publication [2]. On the other hand, the excessive reduction of the RC can lead the blades to be cut into the inner surface of the stator shell and cause the emergency engine failure. Therefore, the task of obtaining reliable information about the value of the RC in the flow part of the gas turbine on a running engine is relevant and is the reason for the long-term interest of gas turbine developers in the appropriate measurement tools [1,5,6,7,8,9].

Despite the existing variety of methods for measuring the RC [5,8], their implementation is limited by very harsh and even extreme conditions in the gas-air path, which are associated with high temperatures in the measurement zone, reaching 1200 °C or more in GTE turbines, high linear speeds of the monitored blades tips, contamination of the gas-air path by fuel combustion products, vibrations, etc. The measurement methods on the base of eddy current sensors are one of the most promising methods for obtaining information about RC in such extreme conditions [10,11,12,13,14,15,16].

The single-coil eddy current sensors (SCECS) being developed in the Institute for the control of complex systems of the Russian Academy of Sciences [17] constitute a separate and independent branch among the eddy current sensors. The SCECS sensing element (SE) is a single current loop. The plane of the SE contour can be oriented both parallel and orthogonally in the direction of the monitored object.

The descriptions of the original constructions of the SCECS used for measuring of the RC in the GTE are given in works [17,18,19]. The SE of the sensor is a segment of a linear conductor that is connected using current leads and a volume coil of the matching transformer (MT) to the measuring circuit (MC). The SE is inserted into the flow part of the compressor or turbine through the mounting hole in the stator. The MT is placed on the outside of the stator shell in favorable temperature conditions. The interaction of the SE and the blade tip provides measurement information about the RC.

The computerized measurement systems were built on the basis of the SCECS with a SE in the form of a conductor segment. The systems were used in GTE bench tests to monitor the RC in various operating modes of power plants. The measurement systems provided engine developers with documented data on the value of the RC between GTE movable and fixed structural elements, including information about the RC for each blade of the impeller of the monitored stage of the compressor and turbine of the GTE. The systems performance, metrological consistency and reliability were confirmed during the testing processes. The total operating time of the measurement systems was more than a hundred hours [17,20].

In real conditions the blade tips perform complex multi-coordinate displacements associated with the bending and twisting of the blade body, the axial displacement of the rotor in a radial thrust bearing, etc. The inductance of the primary MT winding of the SCECS contains all information about the coordinates of the blade tips offset and this information can be extracted using the so-called cluster measurement methods [20,21]. The methods involve the concentration of a cluster (group) of SCECS in the measurement zone. The SE of the sensors have a certain orientation relative to each other and the reference system. The number of the sensors is determined by the number of the measured coordinates. The inductances of the primary windings of the MT of the sensors are fixed at the moment when the blades roots pass the selected reference system. The desired coordinates are calculated using the pre-obtained calibration characteristics (CC) of each sensor during the processing of the received data. In particular, the axial displacements of the shafts in radial thrust bearings can be estimated in addition to the measured RC. And this, in turn, allows to identify the initial stage of the intensive wear of the friction pair.

The detection of metal wear particles in the lubrication systems of the bearing units of the power plants is another application of the SCECS. Stepanov [22] states that the total number of GTE failures associated with friction nodes can exceed 30%. It is obvious that the timely detection of wear particles in the oil of the GTE lubrication system eliminates the emergency situations associated with the premature bearing failures. The SCECS with orthogonal orientations of the SE contour to the monitored object (a cluster of SCECS) can be used for construction of in-line debris detectors for the GTE oil systems of different diameters. They can determine the size of the metal particle and identify its magnetic properties.

The article provides an overview of the existing and newly developed methods and tools for monitoring dangerous states of the GTE on the base of SCECS. The description of the design solutions of the SCECS is given and the principles of converting its information parameter in MC are considered. Attention is focused on the problems associated with various constraints that make it difficult to implement the methods and create workable and effective monitoring tools. The possible solutions are proposed. Examples of practical application of the SCECS and the systems on their basis in bench tests of GTE, their units and aggregates are given.

## 2. Radial Clearance Single-Coil Eddy Current Sensor with a Sensing Element in the Form of a Conductor Segment

Receiving the information about the displacements of GTE structural elements, including those that form the RC, occurs directly in the flow part of the gas-air path of the compressor and turbine in a limited space. If we take into account that the linear speed of the blade tips may exceed the speed of sound, then the time for each blade of the impeller to pass under the SE, and, consequently, the measurement time for each blade, may be very small and will be counted in several microseconds. Meanwhile, the measurements are made in conditions of high temperature more than 600 °C in the compressor and over 1200 °C in the turbine, accompanied by a high level of vibration and pollution of the gas-air tract. All these conditions are very harsh and even extreme. Measurements under such conditions are a non-trivial task, which can be solved by using the SCECS with a SE in the form of a segment of a linear conductor [17,18,19].

**Figure 1:**
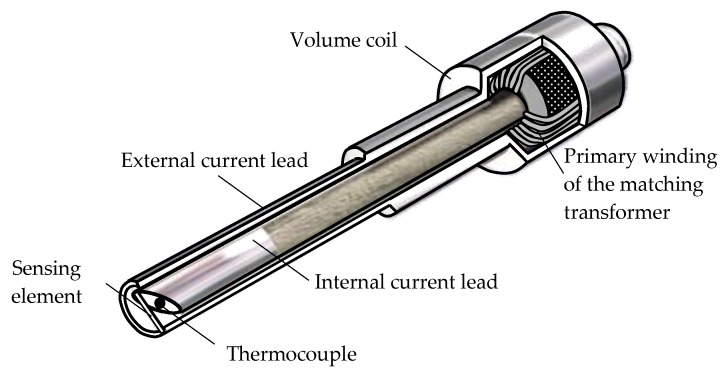
Schematic image of a typical SCECS with SE in the form of a conductor segment.

Although currently a large number of SCECS varieties exist for measuring the RC, they are all structurally identical. The schematic image of a typical SCECS with SE in the form of a conductor segment is shown in Figure 1.

The performance of the sensor in the flow part of the compressor and turbine of the GTE is provided, first of all, due to the simplest design of the SE, made as a segment of the conductor with a square or rectangular cross-section. The SE is made of the same grades of heat-resistant steel as the blades. Non-inductive (in the first approximation) current leads, made of the same material, in the form of two coaxial cylinders, pass through the mounting hole and connect the SE to the MT located on the outside of the stator shell. The primary MT winding is included in the MC with pulsed supply. The secondary MT winding is a “volume coil” that is electrically connected to the current leads and the SE. Near the SE in the end part of the internal current lead a hot junction of a thermocouple is placed. The thermocouple monitors the changes in the temperature of the SE and is intended for use in the thermal correction channel of the RC measurement system.

**Figure 2:**
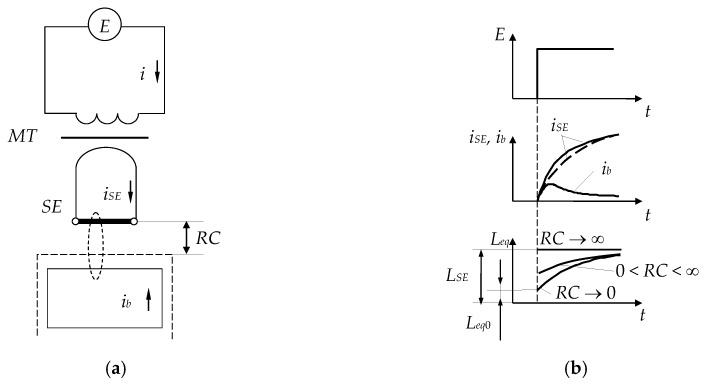
Electrical configuration of the SCECS (**a**) and the time diagrams of changes in the currents and equivalent inductance of the SE (**b**).

The electrical configuration of the SCECS and the time diagrams of changes in the currents and equivalent inductance of the SE as a result of its electromagnetic interaction with the blade tip are shown in Figure 2.

Let’s assume that the MT does not distort the front edge of the supply voltage of a rectangular shape, which excites the *i_SE_* current in the SE circuit. If the blade is at a large distance from the SE (*RC* → ∞) and the influence of eddy currents in the SE associated with the magnetic field caused by the current *i_SE_* can be neglected, the current *i_SE_* is determined only by the inductance *L_SE_* and the ohmic resistance of the SE (*R_SE_*). In this case the *i_SE_* changes in time will have an exponential growing character (Figure 2b, dashed curve). With the approach of the blade tip (*RC* → 0), the eddy currents appear in the blade under the action of a magnetic field created by *i_SE_* and time-varying *i_b_* current appears in the contour that imitates the blade (Figure 2a). The current *i_b_*(*t*) affects the resulting magnetic field and this leads to changes in the shape of the current *i_SE_*(*t*) and its deviation from the exponential dependence (Figure 2b, solid curve). Such deviation can be interpreted as the influence of a time-variable equivalent inductance *L_eq_*(t), whose instability in the transition mode is explained by the influence of eddy current *i_b_*.

Sekisov et al. [17] show that at the beginning of the transition process at *t* → 0, the equivalent inductance is minimal and depends on the RC:

(1) $$L_{eq}=L_{eq0}=\underset{t\to 0}{lim}\left[L_{SE}-M\frac{i_{b}}{i_{SE}}\right],$$

where *M* is the mutual inductance of the circuits of currents *i_SE_* and *i_b_*. At *t* → ∞, the eddy currents are attenuated (*i_b_* = 0) and the inductance *L_eq_* increases and tends to the *L_SE_* (Figure 2b). Obviously, the beginning of the transition process is characterized by the greatest sensitivity of the inductance *L_eq_*_0_ to the RC changes and in this connection the time point *t* = 0 is the most attractive for subsequent transformations. Similar results were obtained on numerical models of the electromagnetic interaction of an idealized SE with a blade simulator in the form of a flat rectangular plate and even with a complex-shaped simulator corresponding to the actual existing blades with their geometric and electrophysical parameters [23,24,25].

Therefore, the method of the first derivative [17] is preferable for converting of the changes of the SE equivalent inductance (*L_eq_*). The method provides for fixation of the derivative of the current *i* in the MT primary winding at the time of *t* → 0. It is assumed that the inductance of the primary winding $L=n^{2}L_{eq}$, where *n* is the MT transformer ratio (n=w1w2, *w*_1_ and *w*_2_—the number of turns, and *w*_2_ = 1) and its ohmic resistance is *R* [17]. If we do not take into account the self-capacity of the MT primary winding, then the transition process in its circuit with pulsed supply *E* and the time-varying of *L_eq_*(*t*) (and consequently *L*(*t*)) is described by the Equation:

(2) $$L\left(t\right)\frac{di}{dt}+i\cdot \left[\frac{dL\left(t\right)}{dt}+R\right]=E.$$

The current derivative at the time of the occurrence of the *E* voltage can be found without solving the Equation (2) since *i* = 0 at *t* = 0 and, therefore ${\frac{di}{dt}|}_{t=0}=\frac{E}{L\left(0\right)}$, where *L*(0) is the inductance at *t* = 0 ($L\left(0\right)=w_{1}^{2}L_{eq0}$). This means that the current derivative does not depend on the resistance *R* and is determined by the instantaneous value of the inductance. Thus, the method of the first derivative provides the minimum conversion time, the maximum sensitivity to RC at the time of *t* → 0 and an increased accuracy by eliminating the influence of *R* changes.

**Figure 3:**
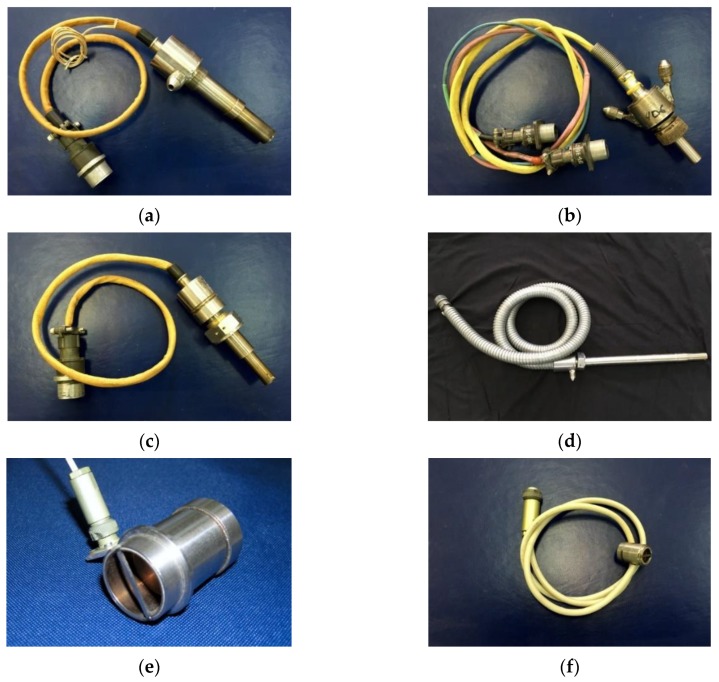
Examples of SCECS for RC measuring in a GTE turbine with forced air (**a**) and liquid (**b**) cooling; in a GTE compressor (**c**); in shaft seals (**d**); in a ducted propfan (**e**) and in GTE support bearings (**f**).

Some examples of the SCECS for RC measuring in various elements of the GTE are shown in Figure 3.

To reduce the influence of the temperature effects on the SCECS components the additional witness SCECS is used. The SE of the witness SCECS is inserted through an additional mounting hole into the wheel space so that the temperature conditions are identical to the conditions of the SE of the working SCECS and there is no electromagnetic interaction with the blade tips. For this purpose, the witness SCECS is shifted from the working SCECS to 1/2 blade pitch of the monitored compressor or turbine rotor wheel. The witness SCECS is connected to the general MC with the working SCECS, where it performs compensation functions. This method was widely used in experimental studies of gas turbine engines under bench conditions [17,20].

However, there are certain difficulties in selecting a pair of SCECS with identical parameters that are related to the sensor manufacturing technology limitations. Moreover, the use of additional SCECS requires additional mounting holes on the stator, which is not always desirable, and in some cases is unacceptable. A RC measuring method without using an additional witness SCECS is described by Belopukhov et al. [26]. Compensation of temperature effects on the sensor is achieved by measuring extreme values of the equivalent inductance of the MT primary winding of the same SCECS, which depends on the RC and temperature when a monitored blade passes by the SE and only on temperature when a blade-to-blade gap passes by the SE, with the subsequent calculation of the difference of the measured values, which is determined only by the RC.

However, even in this case, only circuitry solutions fail to achieve full compensation for the influence of the temperature effects on the SCECS. Podlypnov [27] shows that the extreme equivalent inductances of the MT primary winding of SCECS are also significantly affected by the temperature of not only the monitored, but also adjacent blades. Therefore, an algorithmic method for correcting the temperature effects on SCECS is additionally used in the GTE bench tests [17].

The method provides for using the families of the CC of measuring channels in the form of dependencies of ADC codes at the output of the MC with SCECS on the RC and the temperature. The families of CC are preliminary obtained by an experimental way on the specialized stands (calibration devices [17]). The obtained CC are loaded into the memory of the measurement system and then are used in data processing during the system operation to calculate the desired RC taking account of the temperature in the gas-air path of the GTE. To implement this method, the SCECS is additionally equipped with a thermocouple, which hot junction is placed near the SE and provides a temperature measurement in the area where the SE is located (Figure 1).

## 3. Clusters of SCECS and Their Use for Measuring the Displacements of the Blade Tips on Several Coordinates

**Figure 4:**
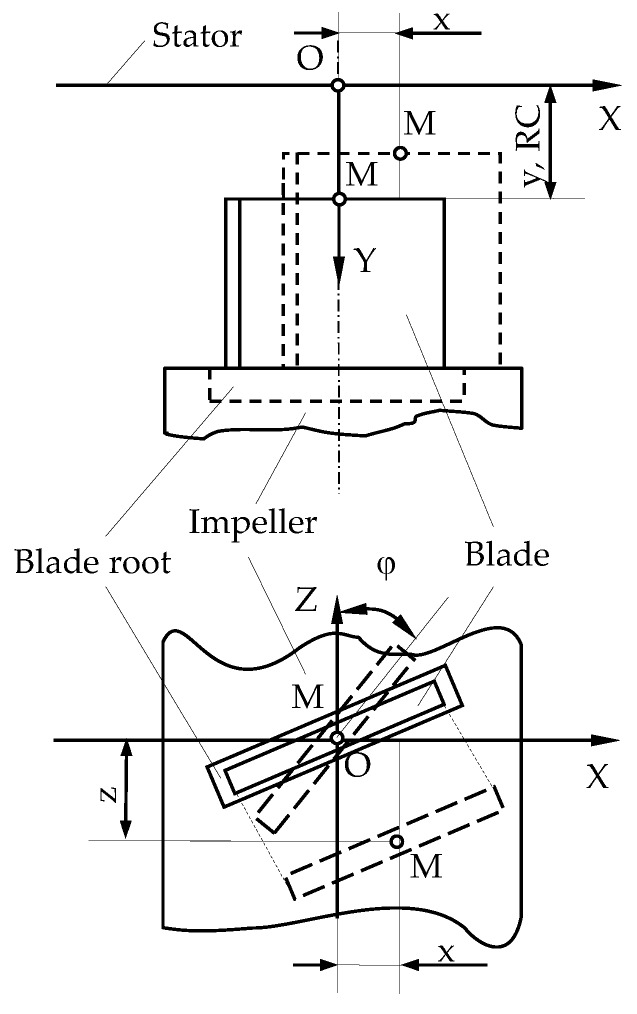
Offset of the blade tips in the OXYZ reference system.

The tips of the compressor and turbine blades perform a complex multidimensional movement in real conditions of GTE operation. These movements are caused by factors of different physical nature. For example, the temperature, elastic and plastic deformations of the rotor structure elements (including the deformations of the blades themselves) caused the radial displacements of the blade tips and are one of the main reasons of RC changing. Thermal elongation of the shaft and the displacement of the rotor wheel in radial thrust bearings due to the aerodynamical loads are the reasons of blade tips displacement in the direction of the GTE axis. Aerodynamic loads also lead to bending of the blade pen, etc. Therefore, it can be argued that in GTE operating mode the displacement of the material point (M) selected at the blade tip has a fundamentally vector character and is determined by several coordinates in the Cartesian reference system OXYZ, the center of which (point O) is rigidly attached to the stator at the SCECS position (Figure 4).

In Figure 4 the position of the monitored blade is fixed at the time when the material point *M* on the blade tip is located on the Y coordinate axis of the reference system, the origin of which is determined by point O on the surface of the stator. Offsets of the point *M* are possible in all three linear directions of the coordinate axes: radial (along the Y axis), axial (along the X axis) and in the direction of the rotor rotation (along the Z axis). These movements have a significant impact on the output signal of the SCECS, which integrally contains information about all coordinates of the monitored blade tips displacements. So, on the one hand, the components of blade tips movements in the axial direction and in the direction of the blade wheel rotation are an interfering factor that makes it difficult to obtain information about the RC. On the other hand, they are a source of important information about the processes occurring in the GTE. For example, the destruction of the radial thrust bearing can be determined on the basis of the monitoring of axial displacements of the blade tips [28]. The monitoring of the bending movements along the X and Z axes allows to detect vibrations of the blade tips and diagnose the compressor stall and the pre-surge state of the GTE [29,30,31,32].

Measuring the blade tips displacements along several coordinates is provided by using the methods on the basis of the clusters of SCECS (groups of identical sensors) which SE are oriented in a certain way toward the blade tips and the number of sensors in the cluster corresponds to the number of monitored coordinates. These methods were called “cluster methods” in the works [20,21].

**Figure 5:**
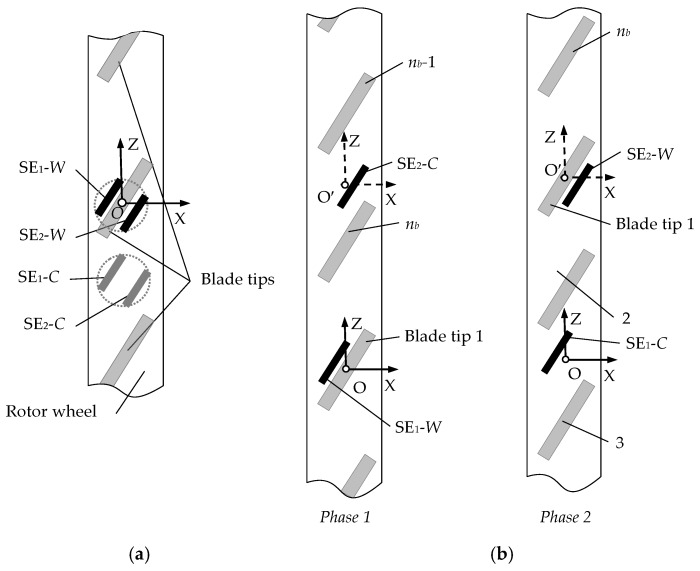
Sensors locations in “concentrated” (**a**) and “distributed” (**b**) clusters of SCECS.

Figure 5a illustrates the method for measuring the axial (x) and radial (y) displacements of the blade tips using the “concentrated cluster” of SCECS. The cluster contains two working SCECS (they are shown in Figure 5a by their sensitive elements SE_1_-W and SE_2_-W) placed very locally to the observation point on the stator (point O is the origin and the geometric center (g.c.) of the cluster). To compensate for the temperature effects on the sensors the same witness (compensation) SCECS are used (in Figure 5a they are also represented by their sensitive elements SE_1_-C and SE_2_-C). The witness SCECS are shifted around the circumference of the stator by a distance multiple of 1/2 blade pitch and have a differential connection to the MC. The fixation of digital codes corresponding to the inductances of the SE_1_-W and SE_2_-W and depending on changes in the coordinates of the blade tips displacements is carried out simultaneously at the moments when the root of each blade passes the g.c. of the cluster (point O).

An obvious limitation of the concentrated cluster of the SCECS is the need to make several mounting holes in the stator shell on a relatively small area. This often causes a negative reaction from the engine developers growing up with an increasing the number of monitored coordinates and the corresponding number of sensors in the cluster (especially since the mounting holes are doubled due to the use of witness (compensation) SCECS). The use of a “distributed cluster” of SCECS allows to eliminate the excessive concentration of SCECS and mounting holes while reducing the number of the sensors.

The location of the distributed cluster with two SCECS intended for the measuring the same (x, y) coordinates is presented in Figure 5b where the sensors are shown by their sensitive elements SE_1_ and SE_2_. The SE_1_ is in the same position as in concentrated cluster. The SE_2_ is equidistantly shifted at an angle 1.5⋅Δψ_b_, where Δψ_b_ is the angular pitch of the blades on the impeller (this corresponds to the shift of the g.c. and the reference system to the distance OO′). The x,y-coordinates are measured in two phases. At phase 1 the root of the blade 1 passes g.c. (point O) and the SE_1_ performs the working functions (SE_1_-W) and the SE_2_ performs the compensation functions (SE_2_-C). At the phase 2 the root of the blade 1 passes the point O′ and the SE_1_ reverses its functions from working to compensation (SE_1_-C) and the SE_2_ vice versa—from compensation to working functions (SE_2_-W). The fixation of digital codes corresponding to the inductances of SE_1_-W and SE_2_-W is carried out at the moments when the root of each blade passes points O and O′ consequently.

In the general case, the use of the concentrated or distributed clusters requires a synchronization of the sensors sampling with the position of the blade wheel in relation to the selected reference system. For these purposes the standard RPM sensors are usually used, but it is also possible to use the SCECS [33]. For the distributed cluster of SCECS the asynchronous with the blade wheel rotation sampling is possible too. In this case, the frequency of the pulsed power supply of the SCECS is selected constant and sufficient to obtain at least 10 samples when the monitored blade tip passes the SCECS sensitivity zone. The code value corresponding to the minimum of the SE-*W* inductance is selected as the information value.

And, finally, when the number of allowed mounting holes in the stator shell is less than the number of monitored coordinates and the corresponding number of SCECS in the cluster, so-called an “incomplete cluster” of the SCECS is used. In the “incomplete cluster” the “unmeasured” coordinates are calculated using specially developed real-time blades behavior models based on the current parameters of the engine regime and its environment [34,35]. For example, the use of an “incomplete cluster” of SCECS in combination with on-line modeling of the blade bend in the system for the RC measuring between the propeller blade tips and the stator shell of the ducted propfan made it possible to reduce the number of SCECS to one sensor at each control point on a stator shell [36].

## 4. Cluster Single-Coil Eddy Current Sensor for Debris Monitoring

There are various methods for the diagnosis of the bearing mount assemblies state. Generally, they all boil down to the detecting of metal wear particles in the engine lubrication system [22]. The methods based on eddy current sensors are the most interesting among the known methods [22,37] for on-board application. The sensor contains the inductance coil that cover the pipeline with the oil stream and creates in its cross section an electromagnetic field interacting with a metal particle. During the electromagnetic interaction the metal particle changes the value of the sensor’s information parameter, which depends on the size and material (ferromagnetic or non-ferromagnetic) of the particle. The Metalscan MS1000 and MS4000 (GasTOPS, Ottawa, Canada) [38,39] are the good examples of such sensors.

**Figure 6:**
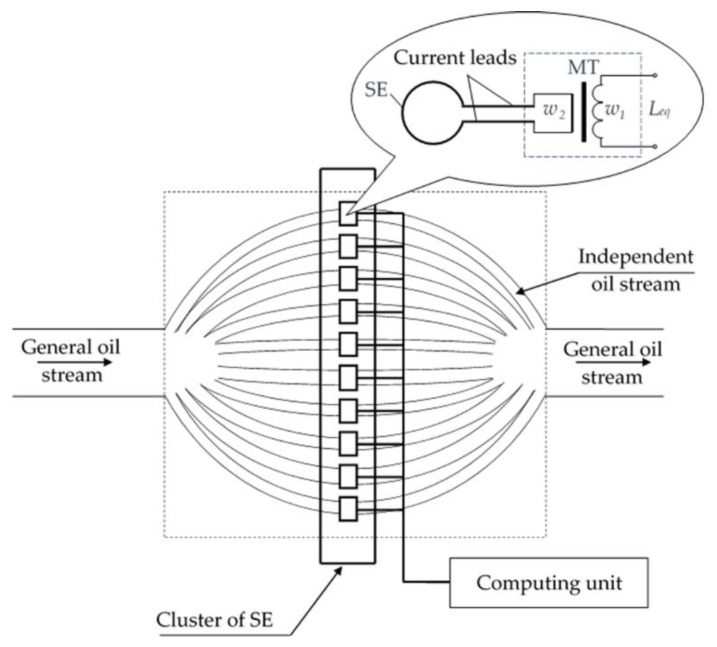
Splitting of the general oil stream into N independent streams and placing the single-coil eddy current SE in them.

However, as shown by Belosludtsev [40], the sensitivity of such sensors decreases as the diameter of the lubrication system pipeline increases, and under certain conditions may not be enough to detect small metal particles. An alternative is a cluster SCECS with an orthogonal orientation of the SE contour to the monitored object (metal particle) [40,41]. For greater sensitivity the general oil stream on the input of the sensor is split into N independent streams with a smaller cross-section area (Figure 6) [42]. Each independent stream is covered by a single current loop located in the pipeline of the engine oil system.

It should be noted that few metal particles running in the same cross section in pipelines with a single SE can be perceived as a single large particle. This leads to the errors in the interpretation of the obtained results. So, the proposed stream separation method, in addition to increasing the sensitivity, also increases the probability of detecting the single metal particles running in the general stream of the pipeline of the power plant’s oil system.

To convert the equivalent inductance of the MT of the SCECS (*L_eq_*) a differential MC is also used, which provides a zero level of the output signal in the absence of a metal particle in the oil channel of the sensor. For this, a second similar SCECS is additionally installed in the cross section of each independent oil channel. The additional sensor is shifted from the first one with the respect to the axis of the channel by a specified distance *h*.

The output signal of a differential MC with SCECS_1_ and SCECS_2_ can generally be represented as:

(3) $$U_{MC}=E\frac{L_{eq2}-L_{eq1}}{L_{eq1}+L_{eq2}},$$

where *E* is the voltage of the MC power supply, *L_eq_*_1_ and *L_eq_*_2_ are the equivalent inductances of the MT primary windings of the both sensors, respectively.

Considering the identity of the SCECS_1_ and SCECS_2_ parameters in the absence of a metal particle in the sensor sensitivity zone, we can write:

*L_eq_*_1_ = *L_eq_*_2_ = *L*_∞_. (4)

The sequential movement of the metal particle first through the SE_1_ contour and then through the SE_2_ contour, leads to the formation of two consecutive voltage pulses of an opposite polarity at the MC output. The pulse amplitude is determined by the size of the metal particle and its location in the plane of the SE contour, and the polarity is determined by the magnetic properties of the particle metal.

For example, a non-magnetic metal particle reduces the equivalent inductance of the SCECS_1_ MT by Δ*L*_1_ when passing through the SE_1_ contour. Considering Equations (3) and (4), the amplitude value of the MC output voltage at the time moment when the particle is in the plane of SE_1_, can be defined as:

(5) $$U_{MC1}=E\frac{\Delta L_{1}}{2L_{\infty }-\Delta L_{1}}.$$

The equivalent inductance of the SCECS_2_ MT also decreases by Δ*L*_2_ when the same non-magnetic particle passes through the SE_2_ contour:

(6) $$U_{MC2}=E\frac{-\Delta L_{2}}{2L_{\infty }-\Delta L_{2}}.$$

**Figure 7:**
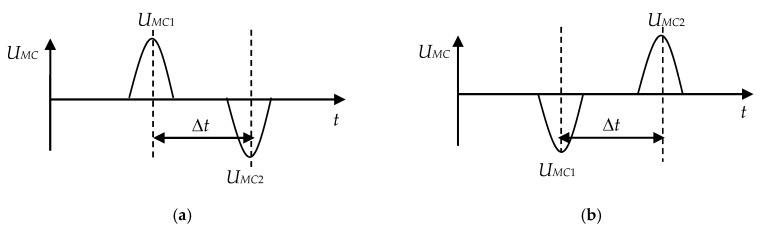
Signal at the output of the differential MC when a non-magnetic (**a**) and magnetic (**b**) metal particles pass the contours of the SE_1_ and the SE_2_.

Therefore, the voltage pulse of a positive polarity is formed at the MC output when a non-magnetic particle passes through the SE_1_ contour and, on the contrary, the polarity of the pulse at the MC output changes from positive to negative when a non-magnetic metal particle passes through the SE_2_ contour (Figure 7a).

A magnetic metal particle increases the SE_1_ and SE_2_ inductances by Δ*L*_1_ and Δ*L*_2_ when passing through the SE contours. In this case the expressions for the amplitude values of the MC output voltages can be written by the analogy with Equations (5) and (6) as:

(7) $$U_{MC1}=E\frac{-\Delta L_{1}}{2L_{\infty }-\Delta L_{2}},$$

(8) $$U_{MC2}=E\frac{\Delta L_{2}}{2L_{\infty }-\Delta L_{2}}.$$

As it can be seen from the expressions Equations (7) and (8), the polarity of the voltage pulses at the MC output also changes. But unlike the case with a non-magnetic metal particle, the pulse polarity changes from negative to positive (Figure 7b) when the magnetic particle passes through the SE_1_ and SE_2_ contours. This allows to identify the material of a particle by its magnetic properties [43].

Setting the two SCECS in the same oil channel at a fixed distance from each other allows also to evaluate the oil flow rate in the oil system of the power plant according to the speed of the metal particle in the sensor’s oil channel. For these purposes the time moments of the appearance of the amplitude values of the MC output voltages (*U_MC_*_1_ and *U_MC_*_2_) are additionally fixed. They correspond to the moments of the sequential passage of the metal particles through the SE_1_ and SE_2_ contours (Figure 7). The oil flow rate is determined by the calculated time interval Δ*t* and the distance *h* between the SE_1_ and SE_2_ [43]:

(9) $$V_{mp}=\frac{h}{\Delta t}.$$

It should be also noted that the use of two SE and the differential MC allows for partial to compensate the influence of some interfering factors (primarily temperature) on the process of transforming the informative parameter and also reduces the number of type II errors associated with the impact of the impulsive noise and the appearance of false signals about the passage of metal particles (the metal particle is fixed only when two pulses of the opposite polarity occurred consistently at the MC output).

As for the determination of particle sizes, their calculation is carried out on the basis of the CC in the form of the dependence of the MC output signal on the position of the particle in the SE contour and the size of the particle. The position of a metal particle in the SE contour during the measurements can be determined through the average oil flow rate, the dimensions of the oil system pipeline and the distribution of elementary flow velocities over the pipeline cross section [44]. It should be noted that in most practical tasks it is enough to estimate the size of the wear particle by assigning it to one of the fixed dimension groups to determine the defect nature in the bearing unit.

## 5. Systems for the Monitoring the Dangerous States of GTE Based on SCECS. Practical Examples

**Figure 8:**
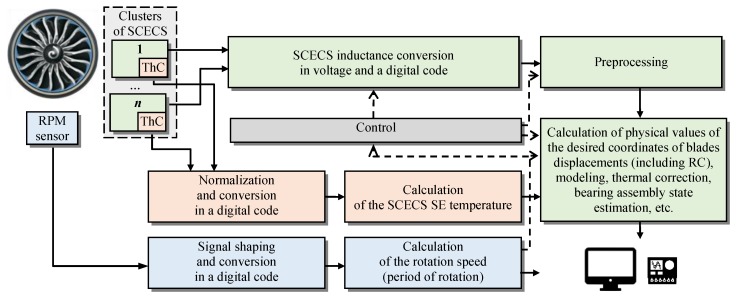
Generalized functional scheme of the system for monitoring the dangerous states of GTE, based on SCECS.

The generalized functional scheme of the system for monitoring the dangerous states of GTE based on SCECS is presented in Figure 8.

Three groups of measuring channels can be defined in the system. They individually or in aggregate provide the required information on the GTE diagnostic parameters (RC, shaft displacements in the bearings, wear particles (debris) of friction pairs, etc.), as well as GTE environment conditions and engine mode parameters.

Data preprocessing is performed after converting the inductances of the SCECS SE to voltage and code. It includes the rejection and data compression, as well as the selection of the informative code values in accordance with the measurement method implemented in the system. If necessary, the information about the ambient temperature in the measurement zone is provided by the thermocouples (ThC) built into the SCECS. The ThC signals are normalized, converted to the code, and then, after temperature calculating, they can be used for algorithmic thermal correction of the measurement results. The signals of the RPM sensor are shaped, converted to a digital code and then used to synchronize the SCECS sampling. They are also a source of information about the power plant operating modes for the on-line simulation of “unmeasured” coordinates in the systems with “incomplete clusters” of SCECS [36,45]. The monitoring results in the form of physical values of the measured parameters are usually displayed on external console devices and are available to the end users. It is also possible to integrate the system based on SCECS with the other diagnostics systems, both as a part of the bench or onboard equipment. In this case, the specific implementation of the functional scheme depends on the tasks to be solved.

Some examples of the systems based on SCECS that were used in the bench tests of GTE, their components and assemblies are given in the Table 1.

Modular crate electronic systems are preferable to be used during the testing of full-size GTE when the studies are carried out at several stages of a compressor or turbine. A 16-channel system for measuring the radial and axial displacements of the blade tips in GTE compressor and turbine (Table 1, line 1) [17,46] is one of the examples of such systems. It was used in bench tests of the Aviadvigatel PS-90 engine and provided the RC simultaneous measurements at four stages (per two stages on the compressor and turbine) in four measurement points in each section. In the compressor only RC were measured and the SCECS on Figure 3c were used. And in the turbine, both RC and the axial displacements of the blade tips were measured with the help of the concentrated cluster of two SCECS (Figure 3a) in each measurement point.

The system had a two-level architecture and implemented parallel-serial processing of the information and data. The lower level contained measuring and microprocessor modules, which were located in the 3U-subtracks. The modules were combined into four microprocessor stations. Each station provided the control of the signals acquisition and conversion from the working SCECS installed on one of the compressor or turbine stages. The overall system control, the processing of the measurement information, its registration and visualization were implemented at the top level in PC.

If studies are carried out at one stage or in one node of GTE and the information volume is not big, then standard remote or built-in PC modules are used. In such systems the SCECS inductance converters are usually built into communication lines from sensors to modules [17,20]. A 2-channel system for RC monitoring in GTE seals (Table 1, line 2) [17] and a 4-channel system for RC measuring and deformation monitoring of the ducted propfan stator (Table 1, line 3) [20,36,45] were built according to this principle.

The system for RC monitoring in GTE seals was used to measure RC between the stator and rotor when testing brush seals on a special high-temperature bench [17]. The RC monitoring was carried out at two points using SCECS presented in Figure 3d. The presence of the subsystems for operational control and continuous data registration were the main features of the measurement system. The first provided the fixation and visualization of the RC measurement results on the operator’s monitor every 1 s. The second was designed to record measurement information on the magnetic media with a period of 750 μs. The subsystems were combined at the level of SCECS and their signal converters. However, they had individual ADC boards built into the PC (Advantech multifunctional boards were used [47]), as well as their own software for measurement information processing.

The RC measurement system in the ducted propfan installation [20,36,45] was designed for bench and on-board tests of the Kuznetsov NK-93 engine [48]. The main objective of the experimental studies (both on the bench and on board) was associated with the monitoring of the dangerous RC between the propeller blade tips and the stator internal surface on a running engine. The system used four SCECS presented in Figure 3e. The sensors were placed in one cross section regularly along the generating line of the stator. This placement of the SCECS also allowed to estimate the deformation of the engine stator shell in real time. For input of analog and discrete signals in a PC the embedded ADC boards of L-Card company were used [49].

A significant limitation of the system was the inability to place more than one SCECS at each control point. In the situation of a wide range of the working angles of turn of the propeller blades and their bending under the influence of aerodynamic loads, the acceptable accuracy of the RC measurements can be obtained by using the “incomplete clusters” of SCECS. For this purpose, the measurement system was “intellectualized” by adding the “on-line” model of the propeller blade bend to the system software. The model provided a real-time calculation of the displacements of the propeller blade tips in the axial direction and in the direction of the propeller rotation. Model simulations then were used to correct the results of the RC measurement. The current information about the propeller rotation speed and the blades angles of turn received from the bench measurement tools were the initial data for the simulation.

It should also be noted that the measurement system had no synchronization of the SCECS sampling with the period of propeller rotation. The association of the RC measurement results with the specific blades was carried out on the basis of a previously obtained “image” of the propfan propeller that takes into account the manufacturing tolerance of the blades linear dimensions.

## 6. Conclusions

The harsh and even extreme conditions for obtaining the information about the GTE structural elements during the engine operation require the use of special measuring instruments. In the authors’ view, the measurement systems based on SCECS and their cluster compositions fall completely under this category. The simplicity of the SCECS design ensures sensors’ functioning at high temperatures in the GTE gas-air path. The algorithms of information processing that implement the appropriate measurement methods make it possible to evaluate a wide range of engine diagnostic parameters, including RC, shaft displacements in the radial thrust bearings, the presence of wear particles of friction pairs in GTE oil system, etc. The performance of the systems was confirmed during experimental studies of full-size GTE and their components. Further research in the direction of the development of SCECS and the systems based on them are aimed at improving the accuracy of measurement, reducing the number of sensors placed on the object and expanding their functional capabilities.

## 7. Patents

Patent RF, no. 2258902, 2005: Method for radial clearances measuring and detecting the blade vibrations of a turbomachine rotor.

Patent RF, no. 2272990, 2006: Method for measuring the multidimensional displacements and detecting the vibrations of the blade tips of a turbomachine rotor.

Patent RF, no. 2273831, 2006: Method for surge detecting and estimating parameters of surge oscillations in compressors of gas turbine plants.

Patent RF, no. 2318185, 2008: Method for radial clearances measuring between the propeller blade tips and the inner surface of the stator shell of a ducted propfan installation.

Patent RF, no. 2344368, 2009: Method for estimating deformations of the propfan stator shell of an aviation gas turbine engine.

Patent RF, no. 2646520, 2018: Method for detecting metal wear particles in the oil stream of a running gas turbine engine.

Patent RF, no. 2668513, 2018: Method for detecting metal particles in the oil of the friction unit lubrication system and determining the oil flow.

Patent RF, no. 2674577, 2018: Method for detecting metal particles in the lubrication system of friction units of power plants with grouping of the particles by size.

## Figures and Tables

**Table 1:** Systems for the monitoring the dangerous states of GTE based on SCECS.

Name and Appearance	Specifications	
16-channel system for RC measuring in GTE [17,46] 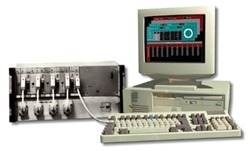	Number of monitored stages: compressor turbine	4 2 2
	Number of measurement points per stage	4
	Number of monitored blades per stage	up to 128
	Rotor speed	600...18,000 rpm
	Permitted range of the turbine wheel axial displacement	±2.5 mm
	Working temperatures: compressor turbine	−40…+650 °C −40…+1200 °C
	RC range	0...3 mm
	RC resolution	0.01 mm
	Reduced measurement error: compressor turbine	< 5% < 10%
2-channel system for RC monitoring in GTE seals [17] 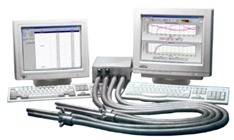	Number of measurement points	2
	Rotor speed	600... rpm
	Working temperatures	−40…+800 °C
	RC range	0...3 mm
	RC resolution	0,01 mm
	Reduced measurement error	< 1%
	RC display rate in operational mode	1 Hz
	RC recording period in continuous inspection mode	750 μs
4-channel system for RC measuring and deformation monitoring of the ducted propfan stator [20,36,45] 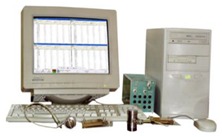	Number of measurement points	4
	Rotor speed	400...1800 rpm
	Propeller blade angles range	15…70 deg
	Working temperatures	−40…+60 °C
	RC range	0...15 mm
	RC resolution	0,01 mm
	Reduced measurement error: 0…2 mm 2…8 mm 8…15 mm	≤ 2% ≤ 5% < 10%

## Abbreviations

ADC	analog to digital converter
CC	calibration characteristic
g.c.	geometric center
GTE	gas turbine engine
MC	measuring circuit
MT	matching transformer
PC	personal computer
RC	radial clearance
RPM	revolutions per minute
SCECS	single-coil eddy current sensor
SE	sensing element
SE-C	compensation sensing element
SE-W	working sensing element
ThC	thermocouple

## References

[1] Danilchenko V.P., Lukachev S.V., Kovylov Y.L., Postnikov A.M., Fedorchenko D.G., Tsybizov Y.I. (2008). Design of Aircraft Gas Turbine Engines.

[2] Melcher K., Kypuros J. (2004). Toward a Fast-Response Active Turbine Tip Clearance Control.

[3] Korson S., Helmicki A.J. An H∞ based controller for a gas turbine clearance control system. Proceedings of the International Conference on Control Applications.

[4] Wiseman M.W., Guo T. An investigation of life extending control techniques for gas turbine engines. Proceedings of the 2001 American Control Conference, (Cat. No.01CH37148).

[5] Lattime S.B., Steinetz B.M. (2004). High-Pressure-Turbine Clearance Control Systems: Current Practices and Future Directions. J. Propuls. Power.

[6] Prokopets A., Revzin B., Rozhkov A. (2004). Necessity for diagnosing radial clearances in the wheel space of gas turbine engines. Gazoturbinnye Tekhnologii.

[7] Kuznetsov N.D., Danilchenko V.P., Reznik V.E. (1991). Control of Radial Clearances in Turbocompressors of Aviation Gas-Turbine Engines.

[8] Inozemtsev A.A., Bazhin S.V., Snitko M.A. (2012). Optimization of the radial clearances of the turboprop engine of an aviation GTE. Vestn. Dvigatelestr..

[9] Kratz J.L., Chapman J.W., Guo T.H. A parametric study of actuator requirements for active turbine tip clearance control of a modern high bypass turbofan engine. Proceedings of the ASME Turbo Expo.

[10] Gerasimov V.G., Klyuev V.V., Shaternikov V.E. (2010). Methods and Devices for Electromagnetic Control.

[11] Flotow A. (2004). Blade-tip monitoring with through-the-case eddy current sensors. Sensors.

[12] Chana K., Lyon D. (2009). Turbo-machinery tip-timing comes of age. Maint. Asset Manag..

[13] Chana K.S., Sridhar V., Singh D. the use of eddy current sensors for the measurement of rotor blade tip timing: Development of a new method based on integration. Proceedings of the ASME Turbo Expo.

[14] Sridhar V., Chana K.S. Tip-clearance measurements on an engine high pressure turbine using an eddy current sensor. Proceedings of the ASME Turbo Expo.

[15] Zhao Z., Liu Z., Lyu Y., Xu X. Verification and Design of High Precision Eddy Current Sensor for Tip Clearance Measurement. Proceedings of the ASME Turbo Expo 2018.

[16] Liu Z., Zhao Z., Lyu Y., Zhao L. (2019). Experimental investigation of inductive sensor characteristic for blade tip clearance measurement at high temperature. Sensors.

[17] Sekisov Y.N., Skobelev O.P., Belenki L.B., Borovik S.Y., Raykov B.K., Slepnev A.V., Tulupova V.V., Sekisov Y.N., Skobelev O.P. (2001). Methods and Tools for Measuring Multidimensional Displacements of Structural Components of Power Plants.

[18] Raykov B.K., Sekisov Y.N., Skobelev O.P., Khritin A.A. (1996). Clearance eddy current sensors with sensitive elements in the form of a segment of a conductor. Devices Control Syst..

[19] Kuteynikova M.M., Raykov B.K., Skobelev O.P. Structural varieties of high-temperature single- coil eddy current sensors. Proceedings of the XIV International conference “Complex Systems: Control and Modelling Problems”.

[20] Belenki L.B., Borovik S.Y., Raykkov B.K., Sekisov Y.N., Skobelev O.P., Tulupova V.V., Skobelev O.P. (2011). Cluster Methods and Tools for Measuring Stator Deformations and Displacement Coordinates of Blade Tips and Blades in Gas Turbine Engines.

[21] Belopukhov V.N., Borovik S.Y., Kuteynikova M.M., Podlypnov P.E., Sekisov Y.N., Skobelev O.P., Skobelev O.P. (2018). Cluster Methods and Tools for Measuring Radial Clearances in Turbine Flow Section.

[22] Stepanov V.A. (2000). Development and Research of Methods and Tools for Complex Diagnostics of Lubricated Friction Units of Gas Turbine Engines by the Parameters of Wear Products in the Oil. Ph.D. Thesis.

[23] Borovik S.Y., Marinina Y.V., Sekisov Y.N. (2007). Model of a cluster single-coil eddy current sensor based on the finite element method. Vestn. SamGTU Ser. Tekhnicheskie Nauk..

[24] Kuteynikova M.M., Sekisov Y.N. Models of electromagnetic interaction of sensitive elements of single- coil eddy current sensors and blades. Status and development. Proceedings of the XIV International Conference “Complex Systems: Control and Modelling Problems”.

[25] Borovik S.Y., Kuteynikova M.M., Podlipnov P.E., Sekisov Y.N., Skobelev O.P. (2015). Modeling the process of measuring radial and axial displacements of complex-shaped blade tips. Optoelectron. Instrum. Data Process..

[26] Belopukhov V.N., Borovik S.Y., Kuteynikova M.M., Podlipnov P.E., Sekisov Y.N., Skobelev O.P. (2018). Method for radial clearance measuring in a gas turbine engine with self-compensation of temperature effects on the sensor. Datchiki Sist..

[27] Podlypnov P.E. (2018). Temperature impact on the controlled and adjacent blades of the compressor impeller when measuring the radial clearances with self-compensation of temperature effects on the sensor. Vestn. SamGTU Ser. Tekhnicheskie Nauk..

[28] Borovik S.Y., Danilchenko V.P., Sekisov Y.N. A vision for using eddy current sensors for diagnostics of thrust bearing of the turbopump of the NK-33 engine. Proceedings of the V All-Russian Scientific and Technical Conference “Actual Problems of Rocket and Space Technology” (V Kozlov Readings).

[29] Borovik S.Y., Raykov B.K., Sekisov Y.N., Skobelev O.P. Methods for measuring and detecting blade vibrations in experimental studies and diagnostics of stall and surge phenomena in compressors of gas turbine engines. Proceedings of the IV International Conference “Complex Systems: Control and Modelling Problems”.

[30] Belkin V.M., Borovik S.Y., Mescheryakov A.A., Raykov B.K., Sekisov Y.N., Skobelev O.P., Tulupova V.V. (2005). Method for Radial Clearances Measuring and Detecting the Blade Vibrations of a Turbomachine Rotor. RF Patent.

[31] Borovik S.Y., Raykov B.K., Sekisov Y.N., Skobelev O.P., Tulupova V.V. (2006). Method for Measuring the Multidimensional Displacements and Detecting the Vibrations of the Blade Tips of a Turbomachine Rotor. RF Patent.

[32] Borovik S.Y., Raykov B.K., Sekisov Y.N., Skobelev O.P. (2006). Method for Surge Detecting and Estimating Parameters of Surge Oscillations in Compressors of Gas Turbine Plants. RF Patent.

[33] Belopukhov V.N., Sekisov Y.N., Skobelev O.P. GTE rotor speed measuring system based on single-coil eddy current sensors. Proceedings of the XIII International Conference “Complex Systems: Control and Modelling Problems”.

[34] Borovik S.Y., Raykov B.K., Sekisov Y.N., Skobelev O.P., Tulupova V.V. Method for measuring the radial displacement of the fan blades using channels of physical and virtual correction. Proceedings of the V International Conference “Complex Systems: Control and Modelling Problems”.

[35] Borovik S.Y., Sekisov Y.N., Skobelev O.P. (2007). Generalized representation of the methods for obtaining measurement information about the coordinates of the displacements of the blades and propellers tips. MekhatronikaAvtom. Upr..

[36] Borovik S.Y., Ignachkov S.M., Il’inskii S.A., Raykov B.K., Sekisov Y.N., Tulupova V.V. (2004). A system of radial clearance measurements in the ducted propfan installation. Izv. Vyss. Uchebnykh Zaved. Aviatsionnaya Tekhnika.

[37] Inozemtsev A.A., Sandratskiy V.L. (2006). Gas-Turbine Engines.

[38] Qualification of an On-Line Bearing and Gear Health Monitoring Technique for in-Service Monitoring of Aircraft Engines and Helicopter Transmissions. http://www.gastopsusa.com/knowledge_center_documents/1/MetalSCAN_ISHM07.pdf.

[39] Haliullin V. (2012). Oil system under continuous control. Permsk. Gazov. Turbiny.

[40] Belosludtsev V.A., Borovik S.Y., Sekisov Y.N., Danilchenko V.P. (2015). Wear diagnostics of aircraft bearings on the base of single-coil eddy-current sensors. Gazoturbinnye Technol..

[41] Borovik S.Y., Sekisov Y.N., Blinov A.V., Muhutdinov F.I. (2017). Transformation of the information in the monitoring system of wear-and-tear particles of friction pairs on the basis of the group of single-coil eddy-current sensitive elements. Turbiny Dieseli.

[42] Borovik S.Y., Korshykov I.G., Sekisov Y.N., Belosludtsev V.A. (2018). Method for Detecting Metal Wear Particles in the Oil Stream of a Running Gas Turbine Engine. RF Patent.

[43] Borovik S.Y., Korshykov I.G., Sekisov Y.N., Belosludtsev V.A. (2018). Method for Detecting Metal Particles in the Oil of the Friction Unit Lubrication System and Determining the Oil Flow. RF Patent.

[44] Borovik S.Y., Korshykov I.G., Belosludtsev V.A., Sekisov Y.N. (2018). Method for Detecting Metal Particles in the Lubrication System of Friction Units of Power Plants with Grouping of the Particles by Size. RF Patent.

[45] Borovik S.Y., Raykov B.K., Tulupova V.V. (2004). System for radial displacements measuring of the fan blade tips. MekhatronikaAvtom. Upr..

[46] Borovik S.Y., Sekisov Y.N., Skobelev O.P., Tulupova V.V., Khritin A.A. (1998). Method and means for measuring radial clearances in gas turbine motors in non-stationary regimes. Avtometriya.

[47] Multifunction. https://www.advantech.com/products/multifunction/sub_1-2mljw2.

[48] NK-93. http://www.airwar.ru/enc/engines/nk-93.html.

[49] L-Card Data Acquisition Devices. https://www.lcard.ru/download/l7xx_users_guide.pdf.

